# Proteomic signatures of in vivo muscle oxidative capacity in healthy adults

**DOI:** 10.1111/acel.13124

**Published:** 2020-03-20

**Authors:** Fatemeh Adelnia, Ceereena Ubaida‐Mohien, Ruin Moaddel, Michelle Shardell, Alexey Lyashkov, Kenneth W. Fishbein, Miguel A. Aon, Richard G. Spencer, Luigi Ferrucci

**Affiliations:** ^1^ Translational Gerontology Branch Intramural Research Program National Institute on Aging National Institutes of Health Baltimore Maryland; ^2^ Vanderbilt University Institute of Imaging Science Vanderbilt University Medical Center Nashville Tennessee; ^3^ Laboratory of Clinical Investigation Intramural Research Program National Institute on Aging, National Institutes of Health Baltimore Maryland

**Keywords:** ^31^P MRS, bioenergetic, mitochondria, proteomic, skeletal muscle

## Abstract

Adequate support of energy for biological activities and during fluctuation of energetic demand is crucial for healthy aging; however, mechanisms for energy decline as well as compensatory mechanisms that counteract such decline remain unclear. We conducted a discovery proteomic study of skeletal muscle in 57 healthy adults (22 women and 35 men; aged 23–87 years) to identify proteins overrepresented and underrepresented with better muscle oxidative capacity, a robust measure of in vivo mitochondrial function*,* independent of age, sex, and physical activity. Muscle oxidative capacity was assessed by ^31^P magnetic resonance spectroscopy postexercise phosphocreatine (PCr) recovery time (τ_PCr_) in the vastus lateralis muscle, with smaller τ_PCr_ values reflecting better oxidative capacity. Of the 4,300 proteins quantified by LC‐MS in muscle biopsies, 253 were significantly overrepresented with better muscle oxidative capacity. Enrichment analysis revealed three major protein clusters: (a) proteins involved in key energetic mitochondrial functions especially complex I of the electron transport chain, tricarboxylic acid (TCA) cycle, fatty acid oxidation, and mitochondrial ABC transporters; (b) spliceosome proteins that regulate mRNA alternative splicing machinery, and (c) proteins involved in translation within mitochondria. Our findings suggest that alternative splicing and mechanisms that modulate mitochondrial protein synthesis are central features of the molecular mechanisms aimed at maintaining mitochondrial function in the face of impairment. Whether these mechanisms are compensatory attempt to counteract the effect of aging on mitochondrial function should be further tested in longitudinal studies.

## Graphical abstract

The skeletal muscle proteins associated with in vivo muscle oxidative capacity, measured by phosphorous magnetic resonance spectroscopy (^31^P MRS), were identified in a cohort of healthy adults. Functional annotation analysis revealed that, independent of age and physical activity, three major protein clusters were strongly associated with in vivo mitochondrial function, namely mRNA processing and splicing, respiratory electron transport chain, and mitochondrial protein translation.

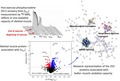

## 1 INTRODUCTION

Among the biological damages and dysfunctions that occur with aging, the reduction of mitochondrial oxidative capacity and consequent effects on energy availability plays a special role (Gonzalez‐Freire, Adelnia, Moaddel, & Ferrucci, 2018a; Lopez‐Otin, Blasco, Partridge, Serrano, & Kroemer, 2013). In fact, decreased mitochondrial oxidative capacity is the only “hallmark” of human aging that has been associated with measures of functional capacity such as muscle strength (Zane et al., 2017) and walking speed (Choi et al., 2016), two robust functional biomarkers that convey information on global health status and risk of disability, frailty, and death in older adults (Guralnik, Ferrucci, Simonsick, Salive, & Wallace, 1995; Studenski et al., 2011).

Muscle mitochondrial function can be assessed reliably and noninvasively by phosphorous magnetic resonance spectroscopy (^31^P MRS) (Arnold, Matthews, & Radda, 1984; Prompers, Wessels, Kemp, & Nicolay, 2014). The time constant of the mono‐exponential function that describes phosphocreatine (PCr) recovery (τ_PCr_) after a short intense exercise provides a well‐established estimate of mitochondrial oxidative phosphorylation capacity (Arnold et al., 1984; Kemp, Ahmad, Nicolay, & Prompers, 2015; Prompers et al., 2014), with smaller values of τ_PCr,_ (more rapid return to baseline PCr levels), reflecting better muscle oxidative capacity. Andreux et al. (2018) demonstrated that τ_PCr_ increases with aging and is smaller in active elderly adults as compared to nonactive or prefrail adults. These authors also reported a reduction of gene expression of mitochondrial genes in prefrail adults, which was consistent with lower in vivo muscle oxidative capacity measured by ^31^P MRS. In agreement with these findings, a comparison of transcriptomics analysis from skeletal muscle biopsies between prefrail and nonfrail older adults found that the 10 most down‐regulated genes in prefrail adults were all mitochondria‐related (Andreux et al., 2018). Finally, our group recently published a discovery proteomic analysis of skeletal muscle aging and found that mitochondrial proteins were the most important class of proteins reduced with aging (Ubaida‐Mohien et al. 2019a). The mechanisms that drive the decline of mitochondrial content and function as well as resilience mechanisms that attempt to offset this decline with aging are still unclear (Ferrucci et al., 2019).

In this work, we conduct a proteomic analysis of skeletal muscle biopsies obtained from healthy individuals dispersed over a wide age range. Muscle oxidative capacity, a robust measure of mitochondrial function, was assessed in vivo by ^31^P MRS. Our aim was to identify proteins that were differentially expressed according to mitochondrial function, independent of age, gender, level of physical activity, and other potential confounders. Based on the set of proteins identified, we make inferences on mechanisms that may drive changes of mitochondrial function both in terms of functional decline or resilience mechanisms aimed at optimizing energy availability and utilization. Overall, we aim to identify new targets and therapeutic strategies for prevention or reversal of the decline in muscle function and mobility with aging.

## 2 RESULTS

### 2.1 Participants characterization

This cross‐sectional study included 57 healthy adults (22 women and 35 men) free of multiple medical conditions, aged 23–87 years who were screened for enrollment through an in‐depth clinical examination performed by trained nurses (see Experimental Procedures section). Summary characteristics of the study participants are reported in Table S1. It is worth noting that this cohort exhibited a wide range of physical activity, with the older adults being slightly more active than the younger ones.

### 2.2 In vivo measurement of muscle oxidative capacity

**Figure 1:**
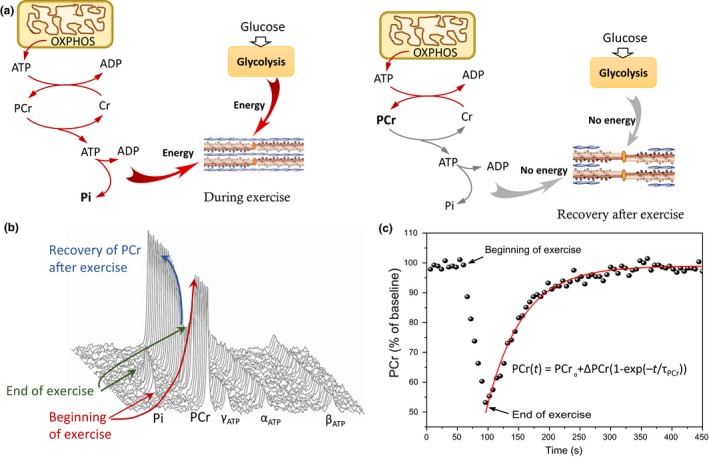
^31^P MRS measurement of in vivo muscle oxidative capacity. (a) Schematic diagram depicting energetic fluxes associated with acute muscle contraction (left) and during postexercise recovery (right). (b) Representative ^31^P spectra collected before, during, and after exercise showing phosphocreatine (PCr) depletion during exercise and concomitant increase in inorganic phosphate (Pi), corresponding to these underlying bioenergetic processes. (c) Time course of PCr changes before, during, and after exercise. Red line corresponds to the fitted mono‐exponential recovery model (see Experimental Procedures section)

Figure 1 illustrates the fundamental principles of in vivo muscle oxidative capacity measurement using the ^31^P MRS technique. In brief, ^31^P MRS records dynamic variations of inorganic phosphorous and organic compounds that contain phosphorus, after a short ballistic knee extension exercise. The outline of energy flux from the mitochondria to the muscle during and after exercise is summarized in Figure 1a. Mitochondria produce energy through oxidative phosphorylation in the form of high‐energy phosphate (HEP) adenosine triphosphate (ATP) (Figure 1a, left panel). The high‐energy phosphate is then transferred to phosphocreatine (PCr) that serves as an energy buffer. When the energy demand increases, PCr splits into creatinine (Cr^+^) and inorganic phosphate (Pi) (Figure 1b) and the released energy is used to anaerobically generate new ATP, which is used by many biological processes, including contraction. This mechanism ensures that large quantities of energy are promptly available at demand, for example, at the beginning of exercise. Upon cessation of exercise, there is a sudden drop in energy demand; however, mitochondria respiratory activity remains high until the baseline level of PCr is reconstituted (see Figure 1b). The postexercise PCr recovery time constant, τ_PCr_, is inversely proportional to the in vivo mitochondrial oxidative capacity, with a smaller time constant reflecting more rapid recovery (Figure 1c) and better oxidative capacity.

### 2.3 Association between skeletal muscle energetic capacity and proteome expression

**Figure 2:**
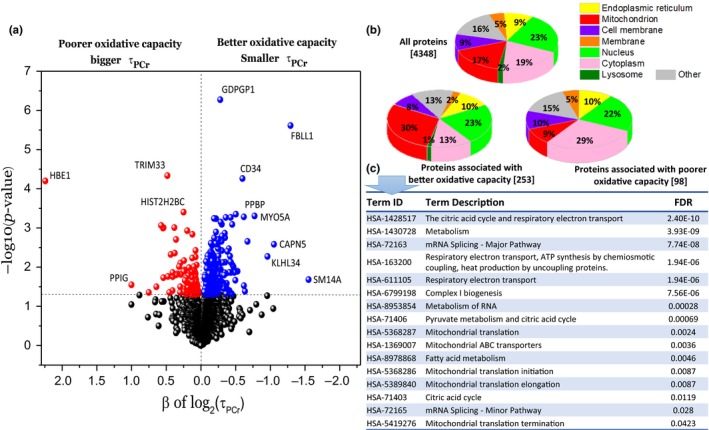
Skeletal muscle proteins associated with in vivo muscle oxidative capacity. (a) Protein association with postexercise phosphocreatine (PCr) recovery time (see statistical analysis in Experimental Procedures section). Blue circles denote proteins that are significantly associated with better muscle oxidative capacity (smaller τ_PCr_), while red circles correspond to proteins significantly associated with poorer oxidative capacity (bigger τ_PCr_). Proteins that did not significantly correlate with τ_PCr_ (*p* > .05) are shown in black. (b) Categorization of all proteins that were quantified using mass spectrometry as a function of their subcellular location (upper pie chart) and categorization of proteins that were significantly associated with τ_PCr,_ that is, significantly associated with better or poorer oxidative capacity (lower pie charts). (c) Top enriched reactome pathways identified by STRING analysis

**Figure 3:**
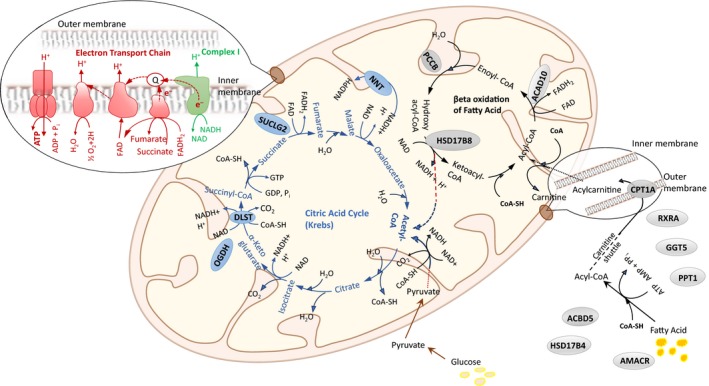
Schematic representation of oxidative phosphorylation pathways involved in muscle bioenergetics. Complex I (green), electron transport chain (red), fatty acid β‐oxidation (gray), and tricarboxylic acid cycle (TCA; blue). Mapping of significant proteins with *p* < .05 are shown in both the TCA and fatty acid cycles. For clarity, complex I and electron transport chain proteins are listed in Table 1

The association of more than 4,300 skeletal muscle proteins with in vivo muscle oxidative capacity as quantified through τ_PCr_ was examined by linear mixed regression models adjusted for age, sex, physical activity, body mass index (BMI), race, and fiber type ratio (see Experimental Procedures section). Greater muscle oxidative capacity, as indicated by smaller τ_PCr_, was associated with increased expression of 253 proteins and decreased expression of 98 proteins (Figure 2a). Overall, 30% and 23% of the proteins linked to better muscle oxidative capacity were mitochondria‐ and nuclear‐encoded, respectively, while only 9% of underrepresented proteins were mitochondria‐encoded (Figure 2b). The proteins found to have increase expression with poorer muscle oxidative capacity were preferentially cytoplasmic. An enrichment analysis indicated that most of these proteins are linked to muscle cell development (Table S2). However, no specific pathway was found within this group of proteins. Therefore, we focused our analysis and discussion on proteins associated with higher skeletal muscle oxidative capacity. Many of these proteins are part of mitochondrial structures or connected with specific mitochondrial functions, including complex I, the electron transport chain (ETC), fatty acid metabolism, and the tricarboxylic acid (TCA) cycle (Figure 2c). A schematic representation of the mechanism of OXPHOS ATP generation is shown in Figure 3, which shows proteins that were significantly associated with better oxidative capacity (smaller τ_PCr_). Many of these proteins were components of complex I, including NADH dehydrogenase 1‐α (NDUFA3, NDUFA7, NDUFA13), NADH dehydrogenase 1‐β (NDUFB7, NDUFB8), NADH dehydrogenase iron‐sulfur protein 4 (NDUFS4), and NADH‐ubiquinone oxidoreductase (MT‐ND1, MT‐ND5; Table 1).

**Table 1:** List of proteins that are significantly (*p* < .05) associated with smaller τ_PCr_ and identified in complex I (CI) and the electron transport chain (ETC). These proteins localize in mitochondria (M) and mitochondrial inner membrane (MIM)

Protein ID	Gene name	Location	Protein Description	*β	*p*	ETC	CI
CIA30_HUMAN	NDUFAF1	M	NADH dehydrogenase 1‐α subcomplex assembly factor 1	−0.274	.031	√	√
COX6C_HUMAN	COX6C	MIM	Cytochrome c oxidase subunit 6C (Cytochrome c oxidase polypeptide VIc)	−0.201	.046	√	
NDUB8_HUMAN	NDUFB8	MIM	NADH dehydrogenase 1‐β subcomplex subunit 8	−0.181	.030	√	√
NU1M_HUMAN	MT‐ND1	MIM	NADH‐ubiquinone oxidoreductase chain 1 (NADH dehydrogenase subunit 1)	−0.178	.007	√	√
NDUS4_HUMAN	NDUFS4	MIM	NADH dehydrogenase iron‐sulfur protein 4	−0.174	.037	√	√
NDUA3_HUMAN	NDUFA3	MIM	NADH dehydrogenase 1‐α subcomplex subunit 3	−0.166	.037	√	√
NU5M_HUMAN	MT‐ND5	MIM	NADH‐ubiquinone oxidoreductase chain 5 (NADH dehydrogenase subunit 5)	−0.139	.020	√	√
NDUA7_HUMAN	NDUFA7	MIM	NADH dehydrogenase 1‐α subcomplex subunit 7 (NADH‐ubiquinone oxidoreductase subunit)	−0.139	.037	√	√
NDUAD_HUMAN	NDUFA13	MIM	NADH dehydrogenase 1‐α subcomplex subunit 13	−0.139	.028	√	√
COX2_HUMAN	MT‐CO2	MIM	Cytochrome c oxidase subunit 2 (Cytochrome c oxidase polypeptide II)	−0.119	.040	√	
NDUB7_HUMAN	NDUFB7	MIM	NADH dehydrogenase 1‐β subcomplex subunit 7 (NADH‐ubiquinone oxidoreductase B18 subunit)	−0.099	.028	√	√
QCR6_HUMAN	UQCRH	MIM	Cytochrome b‐c1 complex subunit 6	−0.087	.021	√	

*β coefficient and *p* were calculated after accounting for all covariates (see Experimental Procedures section). A negative β coefficient indicates a positive correlation with better muscle oxidative capacity (smaller τ_PCr_).

Fatty acid metabolism pathway was also enriched as a function of in vivo muscle oxidative capacity. One of the significant proteins in this pathway was the liver isoform of carnitine O‐palmitoyltransferase 1 (CPT1A, β = −0.25, *p* = .007), located in the mitochondrial outer membrane (Figure 3), which plays an essential role in the uptake of acyl‐CoA activated lipids (Eaton, 2002; Gobin et al., 2003). Of note, the muscle isoform of CPT1 (CPT1B), located in the mitochondrial inner membrane, was also negatively correlated to τ_PCr_ (CPT1B, *β* = −0.09, *p* = .12), but the association was not statistically significant. Acyl‐CoA dehydrogenase proteins (ACAD8, ACAD9, ACAD10), which catalyze the addition of trans double‐bonds between C_2_ (α) and C_3_ (β) in the Acyl‐CoA substrate during the initial step of β‐oxidation (He et al., 2011) (Figure 3), were also associated with better oxidative capacity, although correlation with τ_PCr_ was statistically significant only for ACAD10 (ACAD10, *β* = −0.45, *p* = .05). Additionally, the enzyme estradiol 17‐beta‐hydroxysteroid 8 (HSD17B8), which catalyzes the conversion of hydroxyacyl‐CoA to ketoacyl‐CoA in reaction with NAD (Chen et al., 2009), was associated with τ_PCr_ (HSD17B8, *β* = −0.13, *p* = .04). Notably, peroxisomal proteins involved in fatty acid metabolism were also significantly associated with better oxidative capacity. Examples are peroxisomal multifunctional enzyme type 2 (HSD17B4, *β* = −0.08, *p* = .03) and acyl‐CoA‐binding domain‐containing protein 5 (ACBD5, *β* = −0.12, *p* = .02), which binds medium‐ and long‐chain Acyl‐CoA esters (Nazarko et al., 2014). Figure 3 indicates other proteins significantly associated with fatty acid metabolism.

Several enzymes in the TCA cycle also had increased expression and were significantly associated with smaller τ_PCr_ (Figure 3), particularly succinate‐CoA ligase [GDP‐forming] subunit beta (SUCLG2, *β* = −0.22, *p* = .03) and dihydrolipoyllysine‐residue succinyltransferase component of 2‐oxoglutarate dehydrogenase complex protein (DLST, *β* = −0.13, *p* = .02). Other key enzymatic proteins within the TCA cycle that had nonstatistically significantly increased expression with smaller τ_PCr_ (*p* < .1) were isocitrate dehydrogenase (IDH3G, *β* = −0.09, *p* = .07; IDH2, *β* = −0.13, *p* = .08) and fumarate hydratase (FH, *β* = −0.08, *p* = .08).

**Figure 4:**
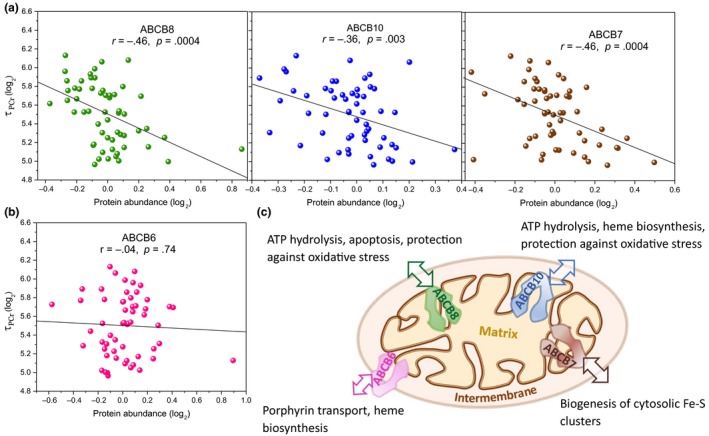
Scatter plot of ATP‐binding cassette sub‐family B (ABC transporters) proteins versus*.* postexercise recovery time of phosphocreatine (τ_PCr_) measured in second. The location of ABC transporters is indicated (a) ABCB8, ABCBA, and ABCB7 in the inner mitochondrial membrane from left to right, respectively, (b) ABCB6 in the outer mitochondrial membrane. Linear regression line of log_2_τ_PCr_ versus*.* log_2_ of protein abundance is shown for the ABC transporters, without adjusting for covariates (It is worth noting that all other statistical results, that is, *p* and β coefficient in the text, are reported after accounting for age and all covariates, see Experimental procedure). (c) Schematic representation of the location of the ABC transporters within mitochondria

A particularly interesting class of proteins that was significantly associated with τ_PCr_ were the ATP‐binding cassette sub‐family B proteins, known as mitochondrial ABC transporter proteins. All four proteins (ABCB6, ABCB7, ABCB8, and ABCB10) from this sub‐family were identified in our analysis (Figure 4), with the proteins located in the inner mitochondrial membrane, ABCB7 (*β* = −0.15, *p* = .02), ABCB8 (*β* = −0.24, *p* = .003), and ABCB10 (*β* = −0.15, *p* = .02), all significantly overrepresented with better oxidative capacity (smaller τ_Pcr_), while no significant difference in expression was observed for ABCB6, located in outer mitochondrial membrane (Figure 4a,b).

Together, these results show that better skeletal muscle oxidative capacity is significantly associated with higher representation of proteins directly involved in mitochondrial energetics (respiration, OXPHOS, TCA cycle, lipid uptake, and oxidation) and substrate transport as indicated by mitochondrial ABC transporter proteins located in the mitochondrial inner membrane.

### 2.4 Overrepresentation of proteins of the mRNA splicing machinery is significantly associated with better muscle oxidative capacity

Several proteins involved in mRNA splicing were also significantly associated with better in vivo mitochondrial oxidative capacity (Figure S1). The greatest degree of upregulation was seen with serine/arginine‐rich splicing factor 9 (SRSF9; *β* = −0.34, *p* = .001), a member of the serine/arginine‐rich family of pre‐mRNA splicing factors that plays an important role in the selection of alternative splice sites during transcription.

Enrichment analysis also unveiled a third significant protein cluster comprising several constituents of the mitochondrial protein translation machinery, including initiation, elongation, and termination. Of the proteins present in this cluster which were significantly associated with τ_PCr_, 39S ribosomal protein L18 (MRPL18, *β* = −0.43, *p* = .01), which facilitates the import of the 5S rRNA ribosome to mitochondria (Smirnov, Entelis, Martin, & Tarassov, 2011), exhibited the strongest association (see also Table S3).

**Figure 5:**
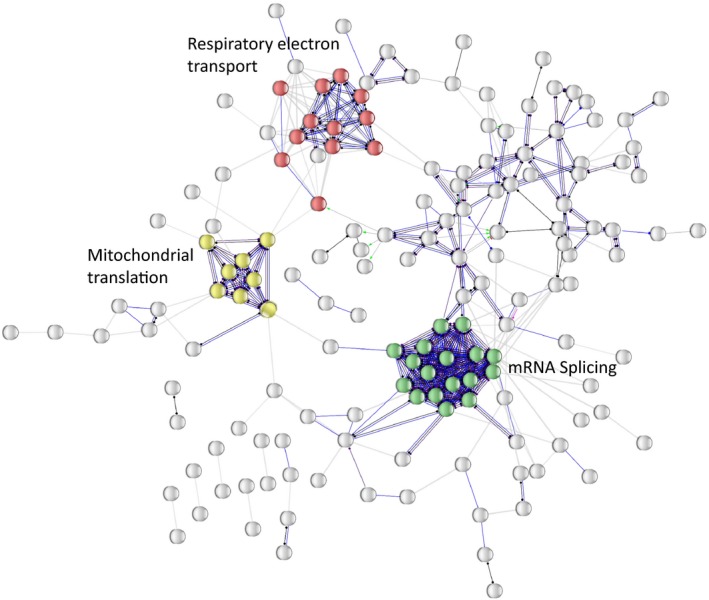
Network representation of the 253 proteins associated with better muscle oxidative capacity as organized by molecular action. Nodes correspond to proteins while edges connecting nodes represent molecular action (e.g., binding, catalysis). STRING network construction was performed using high confidence values (0.7), and the robustness of major protein cluster detection was ascertained by reproducing the results even after allowing for higher number of cluster formation. Color nodes highlight enriched pathways according to reactome analysis, that is, mRNA splicing, respiratory electron transport chain, and mitochondrial protein translation. Nonconnected proteins are not represented

To further ascertain the robustness of our findings, reactome and KEGG pathway analyses were performed on proteins that were significantly overrepresented with better mitochondrial oxidative capacity. Pathways profiled according to molecular action (e.g., binding and catalysis) were in agreement with the enrichment findings; that is, the same protein clusters were found corresponding to mRNA splicing, mitochondrial protein translation and muscle bioenergetics as shown in Figure 5 (see also Figure S2).

Taken together, these findings suggest that overrepresentation of proteins that regulate alternative mRNA splicing and translation in mitochondrial ribosomes, in addition to proteins related to mitochondrial oxidative phosphorylation, may contribute to better oxidative capacity.

## 3 DISCUSSION

In this study, we combined a quantitative discovery proteomics analysis with in vivo measures of mitochondrial oxidative capacity to identify proteins that were differentially represented according to mitochondrial function. Because of the fundamental dependence of mammalian life on adequate mitochondrial function, we searched for proteins patterns that could provide clues about resilience mechanisms aimed at maintaining mitochondrial function in spite of damage accumulation. We elected to focus on healthy adults spanning a wide age range, selected with strict inclusion criteria as being healthy (Adelnia et al., 2019; Ubaida‐Mohien, Lyashkov, et al., 2019b), to allow for a robust assessment of underlying biological mechanisms associated with oxidative capacity independent of the confounding effect of diseases. After adjusting for age, habitual physical activity, sex, race, BMI, and muscle fiber type ratio, a subset of 253 out of 4,300 muscle proteins were significantly associated with better oxidative capacity of skeletal muscle.

Functional annotation analysis revealed three major protein clusters that strongly associated with in vivo mitochondrial function leading to better skeletal muscle oxidative capacity, independent of age, sex, physical activity, and other covariates; these were mRNA splicing machinery, respiratory electron transport chain, and mitochondrial protein translation. Although association of enrichment of proteins from electron transport complexes with better muscle oxidative capacity (i.e., smaller τ_PCr_) was expected (Andreux et al., 2018; Gonzalez‐Freire, Scalzo, et al., 2018b), the enrichment of polypeptides from complex I in the respiratory chain revealed that maintaining the activity of this specific complex may be critical in avoiding decline in oxidative capacity. By inference, and as already suggested by the literature, our data further support the hypothesis that dysregulation of complex I could be involved in the decline of mitochondrial function in skeletal muscle with aging (Aon, Tocchetti, Bhatt, Paolocci, & Cortassa, 2015).

The current study shows that the TCA and β‐oxidation cycles are also significantly associated with better in vivo oxidative capacity. Importantly, our findings suggest that fatty acid oxidation disorder (Vishwanath, 2016) is one of the mechanisms involved in the decline of in vivo muscle oxidative capacity. Consistent with this notion, Bian and colleagues proposed that ACAD10 variation in Pima Indians may increase susceptibility to type 2 diabetes and that this effect may be mediated by impairment of insulin sensitivity via abnormal lipid oxidation (Bian et al., 2010). In addition, in a previous study, we found that in older adults without diabetes, impaired in vivo mitochondrial function (i.e., bigger τ_PCr_ values) is associated with greater insulin resistance and a higher likelihood of prediabetes (Fabbri et al., 2017). Thus, our results confirm those suggestions in the literature that alteration of lipid metabolism and lipid oxidation may affect mitochondrial function, thereby contributing to a wide range of metabolic disorders.

Interestingly, we found that ATP‐binding cassette sub‐family B proteins were significantly associated with better oxidative capacity (smaller τ_PCr_), while a recent study reported no association between of the expression of these proteins and chronological age or the level of habitual physical activity (Ubaida‐Mohien, Lyashkov, et al., 2019b). This finding is particularly important because ABC transporters play major roles both in ATP hydrolysis and in active transport of a large number of substrates (Zutz, Gompf, Schagger, & Tampe, 2009). Structurally, these proteins consist of two transmembrane domains that provide binding sites for transport of a broad range of substrates, and two nucleotide‐binding domains that provide the energy to move substrates across membranes against a concentration gradient (Zutz et al., 2009). In mammals, ABCB6 and ABCB7 have been identified as close homologs of iron‐sulfur cluster transport ATM1 (ATM1‐Yeast) protein which is required for the synthesis of iron‐sulfur proteins (Riedel et al., 2019). ABCB8 and ABCB10 are homologs of ATP‐dependent permease MDL1 and MDL2 protein, respectively (Zutz et al., 2009), but little is known about the physiological function of ABCB8. This close MDL1 homolog is localized in the inner mitochondrial membrane and forms a complex with other mitochondrial proteins including inorganic phosphate carrier, adenine nucleotide translocator, and ATP synthase. The macromolecular complex was identified as a mitochondrial ATP‐sensitive K^+^ channel that seems to be involved in protection of cells against oxidative stress (Ardehali, Chen, Ko, Mejia‐Alvarez, & Marban, 2004); however, the underline mechanism of this reaction is still unclear. On the other hand, it has been demonstrated that overexpression of ABCB10 protein increases heme synthesis and plays a role in cellular protection against oxidative stress during cardiac recovery after ischemic injury (Chloupkova, LeBard, & Koeller, 2003; Liesa et al., 2011). The strong association between τ_PCr_ and ABCB10, which remained significant even after adjusting for possible confounders, suggests that adequate heme synthesis is important for oxygen transport and muscle oxygenation, especially under the high energy demand state of acute muscle contraction.

A major and novel finding of our work is the enrichment of proteins components of the mRNA splicing machinery with better muscle oxidative capacity. Alternative splicing was first described 40 years ago (Alt et al., 1980), revealing that multiple splicing variants can be generated from a single gene, thereby multiplying the protein repertoire that can be generated from a limited number of genes (Nilsen & Graveley, 2010). Previous work showed that the modulation of alternative splicing correlates with ATP depletion in neuronal cells (Maracchioni et al., 2007) and that higher physical activity is linked to decreased expression of proteins that regulate alternative splicing (Ubaida‐Mohien, Lyashkov, et al., 2019b). The mechanistic links between the mRNA splicing machinery with OXPHOS as well as the specific proteins and splicing variants involved in this putative mechanism of metabolic adaptation in skeletal muscle remain unknown. As far as we are aware, our work contributes the first empirical evidence of a possible mechanistic link between alternative mRNA splicing and muscle mitochondria bioenergetics. While we cannot exclude that a progressive deterioration of mRNA splicing machinery (as indicated by lower representation of splicing machinery proteins) is a primary cause of mitochondrial dysfunction, an alternative possibility is that changes in the splicing machinery is a resilience mechanism that regulated energy metabolism flexibility and offsets the decline of mitochondrial function in spite of damage accumulation. This hypothesis should be tested in studies that evaluate changes in skeletal muscle proteomics and in mitochondrial energetics with normal aging and before and after interventions known to affect mitochondrial function, such as physical activity or bed rest.

### 3.1 Limitation of this work

Overall, this work identifies the skeletal muscle proteins that are associated with in vivo oxidative capacity of skeletal muscle independent of several parameters such as age and physical activity. However, changes in protein composition that occur with aging can also be reflected by systematic changes that occur with aging in the representation of different cell types. Of primary concern is the well‐described preferential loss of type II fibers as oppose to type I fibers, because the latter contains more mitochondria and rely on oxidative metabolism. To mitigate, at least in part, this potential source of bias, we adjusted our analyses for the ratio between MYH7 (the myosin of type I fibers) and the sum of MYH1, MYH2 and MYH4 (myosin in type II fibers). In addition, we acknowledge that interferences from other cell types and noncellular matrix that are differentially represented in older adults compared with younger adults may also affect our findings. These include fibroblast, adipocytes, endothelial cells, neurons, intercellular matrix and others. The mass of these alternative cells compared with myofibers is relatively small, their interference is probably limited, and there is currently no established method to offset their contribution in skeletal muscle proteomic analyses.

Finally, our findings were obtained in a cross‐sectional analysis, and therefore, any causal inference between mitochondrial function and proteomic composition remains hypothetical and should considered with caution. Longitudinal studies are needed to test whether alternative splicing and mechanisms that modulate mitochondrial protein synthesis are central features of the resilience molecular mechanisms aimed at maintaining mitochondrial function in the face of damage accumulation.

## 4 CONCLUDING REMARKS

To our knowledge, this is the first study that identifies skeletal muscle proteins associated with in vivo muscle oxidative capacity in a cohort of healthy individuals. Our finding that proteins in the ETC, lipid metabolism, and Krebs cycle were associated with better oxidative capacity provides continuity with previous work. However, the fact that complex I and ATP‐binding cassette sub‐family B proteins located in the mitochondrial inner membrane are particularly overrepresented in individuals with higher oxidative capacity appears to be novel and indicates a potentially important line of further investigation. The association of oxidative capacity with overrepresentation of proteins that belong to the splicing machinery suggests that use of alternative splicing is essential for the maintenance of mitochondrial function. Ultimately, this hypothesis should be confirmed in studies where skeletal muscle proteomics and mitochondrial function is assessed before and after interventions known to affect muscle oxidative capacity, such as increasing physical activity in sedentary individuals or a period of bed rest in previously active individuals.

## 5 EXPERIMENTAL PROCEDURES

### 5.1 Study design and participates

Muscle biopsy and ^31^P MRS data were collected from 57 healthy adults aged 23–87 years (22 women) who participated in longitudinal studies of aging sponsored and conducted by the Intramural Research Program (IRP) of the National Institute on Aging (NIA) under the approval and oversight of the IRB of the National Institute of Environmental Health Science. We used data from 49 subjects from the Genetic and Epigenetic Study of Aging and Laboratory Testing (GESTALT) study and another 8 subjects from the Baltimore Longitudinal Study of Aging. Inclusion criteria for all participants were those of the GESTALT study (Adelnia et al., 2019; Ubaida‐Mohien, Lyashkov, et al., 2019b). Briefly, participants were required to be free of major diseases or cognitive or physical impairment, with the exception of controlled hypertension. Screening was performed through an in‐depth clinical examination performed by trained nurses, following standard protocols, and gave written informed consent at the time of enrollment. Recruited subjects underwent a 3‐day comprehensive examination, including a complete physical examination and health history assessment, interview with standardized questionnaires, ^31^P MRS measurements and muscle biopsy at the NIA IRP Clinical Research Unit. Participants’ characteristics were collected at the same visit.

### 5.2 In vivo muscle oxidative ATP synthesis determined by ^31^P MRS

Phosphorus‐31 nuclear magnetic resonance spectra were collected using a 3T Philips Achieva MR scanner (Philips). Participants were positioned supine and feet first in the bore of the scanner with knees supported at about 120° flexion; each participant was shifted laterally to place the left thigh close to the isocenter of the magnet. A 10‐cm ^31^P‐tuned surface coil (PulseTeq) was fastened above the middle of the left thigh over the vastus lateralis muscle. Thighs and hips were secured with cushions and straps to ensure subject comfort and to minimize the effect of motion during the protocol. A rapid ballistic knee extension exercise was performed by participants for approximately 20–50 s (see below), similar to the protocol employed by Choi et al., 2016 (Choi et al., 2016), while a total of 75 ^31^P NMR spectra were collected continuously before, during and after exercise using a pulse‐acquire surface‐coil localized sequence with the following parameters: TR = 1,500, flip angle 90°, bandwidth = 2,250 Hz, number of samples = 2048, and four averages, resulting in a time resolution of 6 s per spectrum.

Exercise duration was defined to approximately achieve 50% depletion of PCr peak height (at least 30% but not more than 70%) compared with initial baseline values, while avoiding profound intracellular muscle acidosis (defined as pH < 6.8) by monitoring the chemical shift of inorganic phosphate (Pi) peak relative to PCr as a measure of pH (Paganini, Foley, & Meyer, 1997). The average value of PCr depletion overall sample size was 58.3% (±14.5) as indicated in Table S1, and a pH of 6.87 (±0.06). The series of acquired spectra were processed using Java‐based jMRUI software (version 5.2) and quantified using a nonlinear least‐squares algorithm, AMARES (Naressi, Couturier, Castang, Beer, & Graveron‐Demilly, 2001).

The approach utilized here for determining muscle oxidative ATP synthesis rate relies on the consumption of the PCr trough exercise and then allowing a recharging of PCr through mitochondria oxidative phosphorylation after cessation of exercise. Since the creatine kinase reaction is constantly near equilibrium, the rate of postexercise replenishment of PCr reflects the rate of the muscle oxidative ATP synthesis (Arnold et al., 1984; Kemp et al., 2015; Prompers et al., 2014). A mono‐exponential function, PCr(t) = PCr_₀_ + ∆PCr(1−exp(−t/τ_PCr_)), was used to fit the time‐dependent postexercise PCr recovery, where PCr_₀_ is the PCr spectral line area of phosphocreatine at the commencement of recovery (*t* = 0), ∆PCr is the decrease in PCr line area during exercise, and τ_PCr_ is the recovery time constant of the phosphocreatine. τ_PCr_ is inversely proportional to in vivo oxidative capacity of skeletal muscle, that is, oxidative ATP synthesis rate, with negligible contribution from anaerobic metabolism. In vivo muscle oxidative capacity measured using ^31^P MRS recovery rate has been validated against direct mitochondrial respirometry (McCully, Fielding, Evans, Leigh, & Posner, 1993) and demonstrated to be independent of exercise intensity when intracellular pH changes are small (Arnold et al., 1984; Kemp et al., 2015; Prompers et al., 2014).

### 5.3 Muscle biopsy and tissue processing

A region above the vastus lateralis muscle, identified as the mid‐point of a line drawn between the great trochanter and the mid‐patella upper margin, was identified as the site of muscle biopsy. A trained physician performed the muscle biopsy following a standardized protocol as described previously (Gonzalez‐Freire, Scalzo, et al., 2018b; Ubaida‐Mohien, Gonzalez‐Freire, et al., 2019a; Ubaida‐Mohien, Lyashkov, et al., 2019b;). Briefly, using conventional sterile technique and after provision of local anesthetic, a 6‐mm Bergstrom biopsy needle was inserted transcutaneously into the muscle following the introduction of a gauge needle. Muscle samples were obtained under suction after incising with a coaxial blade. The tissue then was cut into small sections, which were snap frozen in liquid nitrogen and subsequently stored at − 80°C until used for analyses.

Protein concentration was determined using a 2‐D Quant Kit (GE Healthcare Life Sciences) following pulverization and sonication of the muscle sample. Detergents and lipids were removed using a methanol/chloroform extraction protocol (Bligh & Dyer, 1959). Proteins were resuspended in urea buffer (8 M urea, 2M thiourea, 150 mM NaCl; Sigma), reduced (50 mM DTT), and alkylated (100 mM iodoacetamide), then diluted 12 times with 50 mM ammonium bicarbonate, and digested for 18 hr at 36°C using a trypsin/LysC mixture (1:50 w/w, Promega). Peptides were desalted and stored at −80°C. TMT labeling was performed according to the manufacturer's guidelines (TMT6plex, Thermo Fisher, Cat# 90066). Each TMT set included one donor randomly selected from each of 5 different age groups (20–34, 35–49, 50–64, 65–79, 80+ years) and one reference sample. Each sample was spiked with 200 fM of bacterial beta‐galactosidase digest (SCIEX) prior to TMT labeling. Labeled peptides were combined and fractionated.

### 5.4 LC‐MS analysis and proteomic informatics

High pH reversed‐phase chromatography with a fraction concatenation approach (Wang et al., 2011) was used to prepare samples prior to liquid chromatography–mass spectroscopy (LC‐MS) analysis. Briefly, tissues samples were separated using an organic gradient (5% to 50% B, 100 min) into 99 fractions and merged into 33 master fractions (fraction 1, 34, 67 = master fraction 1; fraction 2, 35, 68 = master fraction 2 and so on). Combined fractions were speed vacuum dried, desalted, and stored at −80°C until final LC‐MS analysis. Finally, mass spectra were obtained using an UltiMate 3000 Nano LC System coupled to a Q Exactive HF mass spectrometer (Thermo Scientific) with a heated capillary temperature of +280°C and spray voltage set to 2.5 kV. Full MS1 spectra were acquired from 300 to 1,500 m/z at 120,000 resolution and 50 ms maximum accumulation time with automatic gain control set to 3 × 106. Dd‐MS2 spectra were acquired using a dynamic m/z range with fixed first mass of 100 m/z. MS/MS spectra were then resolved to 30,000 with 155 ms of maximum accumulation time and automatic gain control target set to 2 × 105. The 12 most abundant ions were selected for fragmentation using 30% normalized high collision energy. A dynamic exclusion time of 40 s was used to discriminate against the previously analyzed ions.

Data collected from LC‐MS analysis were converted to mascot generic format (.mgf) using MSConvert software (ProteoWizard 3.0.6002) to produce a list of MS ions with retention times and MS/MS spectra. The list of ions was then searched with Mascot 2.4.1 and X!Tandem CYCLONE (2010.12.01.1) using SwissProt Human sequences from the UniProt database (Version Year 2015) to identify possible peptides, as explained elsewhere (Ubaida‐Mohien, Lyashkov, et al., 2019b). A peptide mass tolerance of 20 ppm and 0.08 Da was allowed, according to the known mass accuracy of the instrument. The TMT channels’ isotopic purity was also corrected according to the TMT Kit instructions.

Results from the Mascot and X!Tandem search engines were analyzed by Scaffold Q + 4.4.6 (Proteome Software, Inc), and the peptide and protein probabilities were calculated by PeptideProphet and the ProteinProphet probability model (Keller, Nesvizhskii, Kolker, & Aebersold, 2002; Nesvizhskii, Keller, Kolker, & Aebersold, 2003). Results were filtered at 1% thresholds of both protein and peptide false discovery rate (FDR) and required at least one unique peptide for protein identification. Those proteins that were identified from a single peptide were included in further analysis if that identification was confirmed by more than one search engine and identified across all samples, as previously described (Ubaida‐Mohien, Gonzalez‐Freire, et al., 2019a; Ubaida‐Mohien, Lyashkov, et al., 2019b). The quantitative reporter ion intensity values were extracted from Scaffold and decoy signals, contaminant signals, peptide signals shared between more than one protein were removed. The log2 transformed reporter ion abundance was then normalized by median subtraction from all reporter ion intensity signals belonging to a protein across all channels. Finally, relative protein abundance was estimated by the median of all peptides for a protein combined. Protein sample loading effects were corrected by median polishing, that is, subtracting the channel median from the relative protein abundance estimate across all the channels to have a zero median (Kammers, Cole, Tiengwe, & Ruczinski, 2015; Ubaida‐Mohien, Gonzalez‐Freire, et al., 2019a; Ubaida‐Mohien, Lyashkov, et al., 2019b).

### 5.5 Other covariates

Covariates include sex, self‐reported black or non‐black race, and body mass index (BMI). The fiber type ratio was calculated for each participant and was expressed as the ratio between type I (MYH7) and type II myosin fibers (MYH1, MYH2, MYH4). The level of moderate‐to‐vigorous physical activity (MVPA) was determined using a standard questionnaire as described previously (Ubaida‐Mohien, Gonzalez‐Freire, et al., 2019a; Ubaida‐Mohien, Lyashkov, et al., 2019b). Briefly, participants were asked whether they performed physical activity in the past two weeks. Total participation in each level of physical activity was then calculated by multiplying frequency by amount of time performed in each activity. Finally, the total activity was divided by two, to obtain minutes of MVPA per week, with four categories defied as follows: MVPA < 30 min per week coded as 0, MVPA ≥ 30 and less than 70 min per week coded as 1, MVPA ≥ 75 and less than 150 min per week coded as 2, and MVPA ≥ 150 coded as 3. The resulting ordinal variables from 0 to 3 were used in the analysis as a continuous variable.

### 5.6 Statistical and enrichment analysis

The association between muscle oxidative capacity (τ_PCr_) and the relative abundance of different proteins was calculated using a linear mixed regression model (R statistical software lme4, v1.1. library) that adjusted for age, physical activity, sex, race, BMI, and fiber type ratio. The TMT mass spectrometry batches were added as a random effect. The protein abundances were analyzed and reported as log2 transformed to account for the prevalence of low versus high abundance, thereby compensating for the limited dynamic range of the muscle protein distribution. Fiber type ratio and τ_PCr_ were also log2 transformed, while those covariates that had a small range of variation (e.g., categorical), or exhibited a normal distribution (i.e., physical activity, BMI) were not log transformed. The beta coefficiant of τ_PCr_ was calculated from the above mixed linear regression model for each protein. Statistical significance was defined as *p* < .05, as indicated using the lmerTest package in R Statistical Software. Proteins with a negative beta coefficient were associated with better muscle oxidative capacity (shorter τ_PCr_) and proteins with a positive beta coefficient were associated with poorer muscle oxidative capacity (longer τ_PCr_). Analysis was performed using R packages (3.3.4 version, Team, 2016).

To detect enrichment of certain biological processes and molecular functions involving the 253 proteins that were significantly over represented with better in vivo oxidative phosphorylation (shorter τ_PCr_), an enrichment analysis was run using the Search Tool for Recurring Instances of Neighbouring Genes (STRING) tool (Szklarczyk et al., 2015). The Reactome and KEGG pathway enrichment, functional annotation clustering, and biological processes (GO function) were assessed in STRING. Annotation and localization of quantified proteins was performed based on the UniProt (UniProt 2019) and STRING database (Szklarczyk et al., 2015). A pathway was considered significantly enriched if FDR was < 0.05.

## CONFLICT OF INTEREST

The authors declare that the research was conducted in the absence of any commercial or financial relationships that could be construed as a potential conflict of interest.

## AUTHOR CONTRIBUTIONS

FA and LF designed the study and LF supervised and directed the project. FA, CU, RM, MS, AL, KWF, MAA, RGS, and LF were involved in collecting and/or analyzing the data. FA and LF wrote the manuscript, and all authors have contributed to and approved the final version of the manuscript.

## Data Availability

The mass spectroscopy proteomic‐related data have been deposited in the ProteomeXchange consortium via the PRIDE partner repository with the dataset identifier PXD011967. The 31P MRS data will also be included in the repository and it will be publicly released after the publication of the manuscript.
