# Supplementary material for: Proteomic signatures of in vivo muscle oxidative capacity in healthy adults
Source: Aging Cell. 2020 Mar 20;19(4):e13124. doi: 10.1111/acel.13124 (PMC7189997; doi:10.1111/acel.13124)
Supplement: Supplementary file 1 [file ACEL-19-e13124-s001.docx]

**Supporting information**

**Supplementary Table 1.** Descriptive characteristics of the study sample.

| Characteristic | Mean (SD) |
| --- | --- |
| Number of participants | 57 |
| Age (years) [range] | 54.7 [23-87] |
| Sex, women (%) | 38.6 |
| Race, African-American (%) | 19.3 |
| Height (cm) | 166 (8.4) |
| Weight (kg) | 68.2 (23.9) |
| Body mass index (kg/m²) | 24.4 (6.2) |
| Systolic blood pressure (mm Hg) | 125.5 (12.7) |
| Diastolic blood pressure (mm Hg) | 65.5 (12.0) |
| Physical activity | 2.0 (1.1) |
| Fiber type ratio^†^ | 0.33 (0.01) |
| τ_PCr_^†^ | 5.5 (0.3) |
| τ_PCr (sec)_ | 46.43 (10.4) |
| PCr depletion*(%) | 58.3 (14.5) |
| pH^ǂ^ | 6.87 (0.06) |

^†^The post-exercise phosphocreatine recovery time (τ_PCr_) and fiber type ratio (estimate from the proteomic data, see method section) are reported as log_2_ transformed. *Reduction of phosphocreatine (PCr) peak height after exercise. ^ǂ^ Minimum pH that has been reached after exercise measured using the chemical shift of inorganic phosphate (Pi) relative to PCr.

**Supplementary Table 2.** Top gene ontology (GO) biological processes enriched in 89 proteins that are significantly associated with poorer muscle oxidative capacity

| Term ID | Term description | FDR | Matching proteins |
| --- | --- | --- | --- |
| GO:0055001 | Muscle cell development | 8.9E-04 | BIN1, CASQ1, CFL2, HOMER1, MYOZ1, STAC3, TTN, UCHL1 |
| GO:0055002 | Striated muscle cell development | 2.6E-03 | CASQ1, CFL2, HOMER1, MYOZ1, STAC3, TTN, UCHL1 |
| GO:0006941 | Striated muscle contraction | 9.9E-03 | CACNA2D1, HOMER1, MYLK2, PGAM2, STAC3, TTN |
| GO:0007519 | Skeletal muscle tissue development | 9.9E-03 | CASQ1, CFL2, HOMER1, MYLK2, STAC3, VAMP5 |
| GO:0009056 | Catabolic process | 9.9E-03 | AGL, DAGLB, DDAH1, ERLIN2, GPD1, GSTM3, NPEPPS, PARK7, PGAM2, PGAM4, PGK1, RNH1, RPL29, RPL4, RPL7, RPS8, SMG8, SMPD4, TBC1D17, UCHL1, UFD1L, VPS37A, VTA1 |
| GO:0016051 | Carbohydrate biosynthetic process | 9.9E-03 | AGL, GPD1, IMPAD1, PGAM2, PGAM4, PGK1 |
| GO:0044248 | Cellular catabolic process | 9.9E-03 | AGL, DDAH1, ERLIN2, GSTM3, NPEPPS, PARK7, PGAM2, PGAM4, PGK1, RNH1, RPL29, RPL4, RPL7, RPS8, SMG8, SMPD4, TBC1D17, UCHL1, UFD1L, VPS37A, VTA1 |
| GO:0046689 | Response to mercury ion | 9.9E-03 | AQP1, PARK7, PGAM2 |
| GO:1902514 | Regulation of calcium ion transmembrane transport via high voltage-gated calcium channel | 9.9E-03 | BIN1, CACNA2D1, CACNB1 |

**Supplementary Table 3.** List of proteins from mitochondrial protein translation (initiation, elongation, termination) that significantly (*p* < 0.05) correlate with shorter τ_PCr_

| Protein ID | Gene name | | Protein Description | β* | *p* | Initiation | Elongation | Termination |
| --- | --- | --- | --- | --- | --- | --- | --- | --- |
| RM18_HUMAN | MRPL18 | 39S ribosomal protein L18, mitochondrial | | -0.433 | 0.014 | $\surd$ | $\surd$ | $\surd$ |
| RM16_HUMAN | MRPL16 | 39S ribosomal protein L16, mitochondrial | | -0.264 | 0.020 | $\surd$ | $\surd$ | $\surd$ |
| OXA1L_HUMAN | OXA1L | Mitochondrial inner membrane protein OXA1L | | -0.148 | 0.035 | $\surd$ | $\surd$ | $\surd$ |
| G45IP_HUMAN | GADD45GIP1 | 39S ribosomal protein L59, mitochondrial | | -0.125 | 0.019 | $\surd$ | $\surd$ | $\surd$ |
| EFTU_HUMAN | TUFM | Elongation factor Tu, mitochondrial | | -0.124 | 0.013 |  | $\surd$ |  |
| IF2M_HUMAN | MTIF2 | Translation initiation factor IF-2, mitochondrial | | -0.120 | 0.034 | $\surd$ |  |  |
| RM09_HUMAN | MRPL9 | 39S ribosomal protein L9, mitochondrial | | -0.117 | 0.033 | $\surd$ | $\surd$ | $\surd$ |
| RM15_HUMAN | MRPL15 | 39S ribosomal protein L15, mitochondrial | | -0.107 | 0.019 | $\surd$ | $\surd$ | $\surd$ |

*The β coefficient and *p* were calculated after accounting for age, physical activity and other covariates (see Experimental Procedures section). The negative β coefficient indicates a positive correlation with better muscle oxidative capacity (smaller τ_PCr_).

**
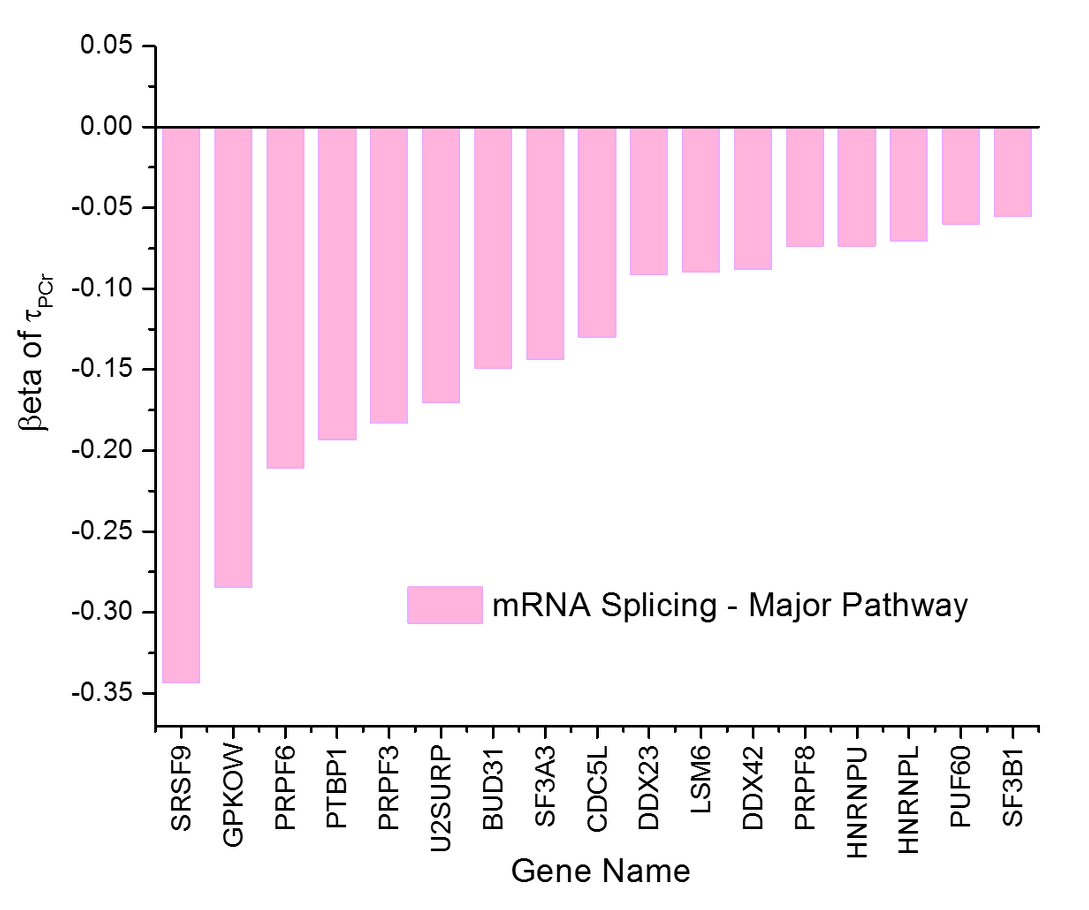
**

**Supplementary Figure 1. Protein components of the spliceosome that correlate significantly (*p* < 0.05) with *in vivo* muscle oxidative capacity (τ_PCr_).** The *x*-axis labels correspond to gene names, and the *y*-axis labels denote the β coefficient obtained from the linear mixed regression analysis after adjusting for age, physical activity and all other covariates (see Experimental Procedures section).

**
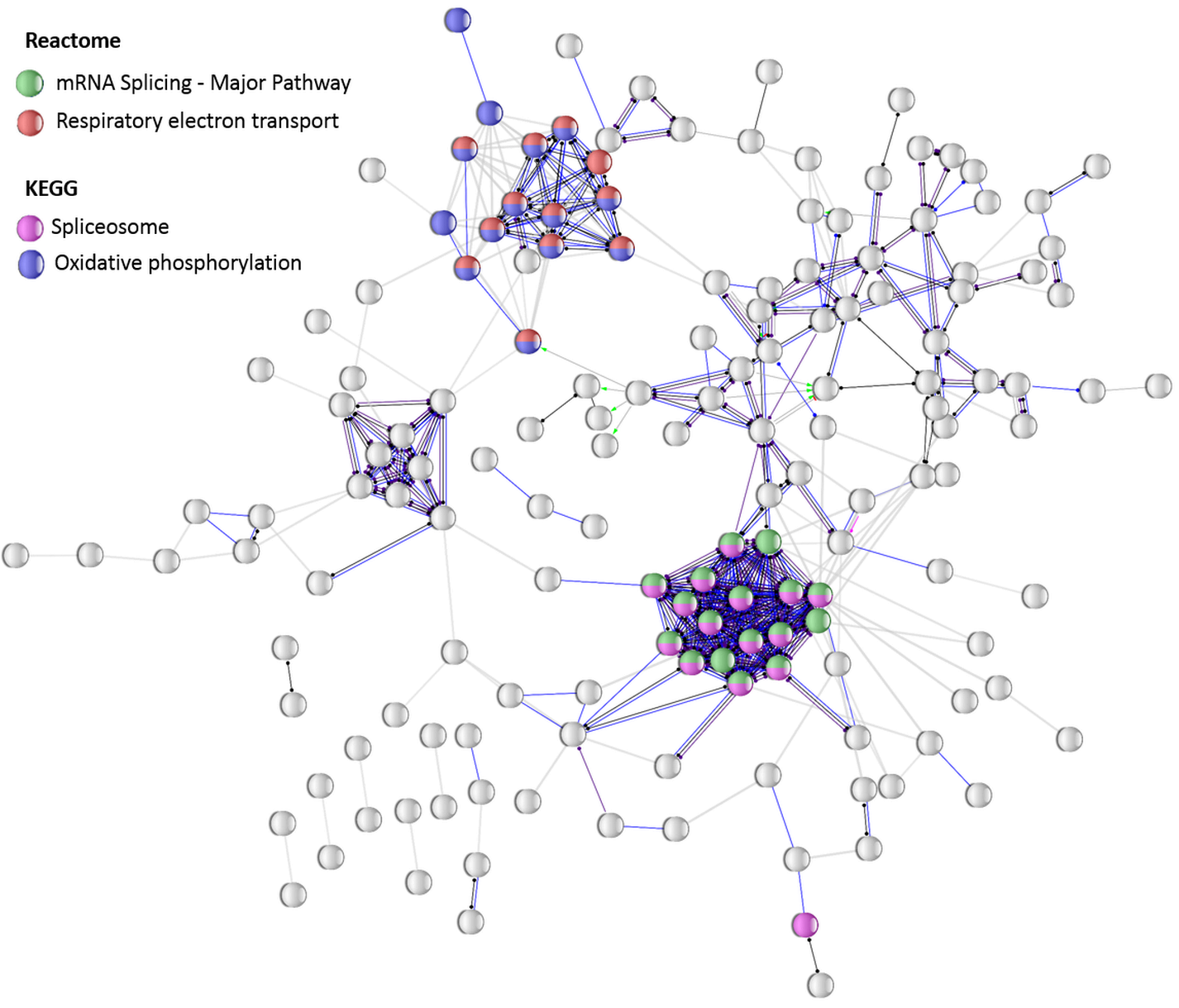
**

**Supplementary Figure 2. Network representation of overrepresented proteins correlating with better muscle oxidative capacity.** Nodes correspond to proteins whereas edges connecting nodes represent proteins’ association to pathways as identified by reactome and KEGG pathways analysis using STRING. Spliceosome (FDR_KEGG_ = 8.4E-09), and OXPHOS (FDR_KEGG_ = 1.95E-07) similar to mRNA Splicing-major pathway (FDR_Reactome_ = 7.7E-08), and respiratory electron transport (FDR_Reactome_ = 7.6E-06), respectively. Non-connected proteins are not represented.
